# FcγR Genetic Variation and HIV-1 Vaccine Efficacy: Context And Considerations

**DOI:** 10.3389/fimmu.2021.788203

**Published:** 2021-12-15

**Authors:** Ria Lassaunière, Caroline T. Tiemessen

**Affiliations:** ^1^ Virus and Microbiological Special Diagnostics, Statens Serum Institut, Copenhagen, Denmark; ^2^ Centre for HIV and STI’s, National Institute for Communicable Diseases, Johannesburg, South Africa; ^3^ Faculty of Health Sciences, University of the Witwatersrand, Johannesburg, South Africa

**Keywords:** FCGR genes, Fc gamma receptor (FcγR), variant, polymorphism, copy number, HIV - human immunodeficiency virus, vaccine, disease progression

## Abstract

Receptors for the crystallisable fragment (Fc) of immunoglobulin (Ig) G, Fcγ receptors (FcγRs), link the humoral and cellular arms of the immune response, providing a diverse armamentarium of antimicrobial effector functions. Findings from HIV-1 vaccine efficacy trials highlight the need for further study of Fc-FcR interactions in understanding what may constitute vaccine-induced protective immunity. These include host genetic correlates identified within the low affinity Fcγ-receptor locus in three HIV-1 efficacy trials – VAX004, RV144, and HVTN 505. This perspective summarizes our present knowledge of FcγR genetics in the context of findings from HIV-1 efficacy trials, and draws on genetic variation described in other contexts, such as mother-to-child HIV-1 transmission and HIV-1 disease progression, to explore the potential contribution of *FcγR* variability in modulating different HIV-1 vaccine efficacy outcomes. Appreciating the complexity and the importance of the collective contribution of variation within the *FCGR* gene locus is important for understanding the role of FcγRs in protection against HIV-1 acquisition.

## Introduction

Despite enormous research efforts over 30 years, a highly efficacious preventative HIV vaccine remains elusive. Nonetheless, each vaccine efficacy trial provided new insight. Only one HIV-1 vaccine trial has shown some level of protection against HIV-1 acquisition. The RV144 vaccine trial (1), conducted in Thailand, achieved modest vaccine efficacy at 31.2%, while 6 other efficacy trials – VAX003 (2), VAX004 (3), HVTN502 (the Step trial) (4), HVTN503 (the Phambili trial) (5), HVTN505 (6), and HVTN702 (the RV144 follow-on trial) (7) – failed to prevent HIV-1 acquisition in vaccinees, and even increased risk in some individuals (4, 8). Many differences could account for the efficacy outcomes, including the vaccine regimen (design, virus subtype, and adjuvant), diversity of circulating virus strains, sex, modes of transmission, different risk populations, geography, and host genetics.

The initial immune correlate analysis from RV144 (9) provided the impetus for more detailed study of immune correlates to better understand vaccine-induced immune protection against HIV-1. These subsequent studies and analyses have revealed the inordinately complex nature of immunological mechanisms that collectively act to provide protection against acquisition of HIV-1 [reviewed in (10)]. In particular, they have highlighted many HIV-specific antibody parameters as correlates of HIV-1 acquisition risk (9, 11–14), many of which bind FcγRs to mediate their functions. Indeed, FcγR-mediated effector functions associate with vaccine protection (9, 15). Host genetic correlates further implicating a role for FcγRs have been identified in three efficacy trials, VAX004 (16), RV144 (17), and HVTN505 (18); each conducted in different population groups with distinct allelic variability across FcγRs (19).

Here we summarize our present knowledge of FcγR genetics in the context of findings from HIV-1 efficacy trials, and include studies of mother-to-child HIV-1 transmission and HIV-1 disease progression. We highlight the complexity of the *FCGR* locus, the importance of using validated methods to aid interpretation, the inclusion of *FCGR* gene copy number determination, and population genetic differences, among other considerations outlined.

## The Low Affinity FcγRs and Host Genetic Variability

**Figure 1:**
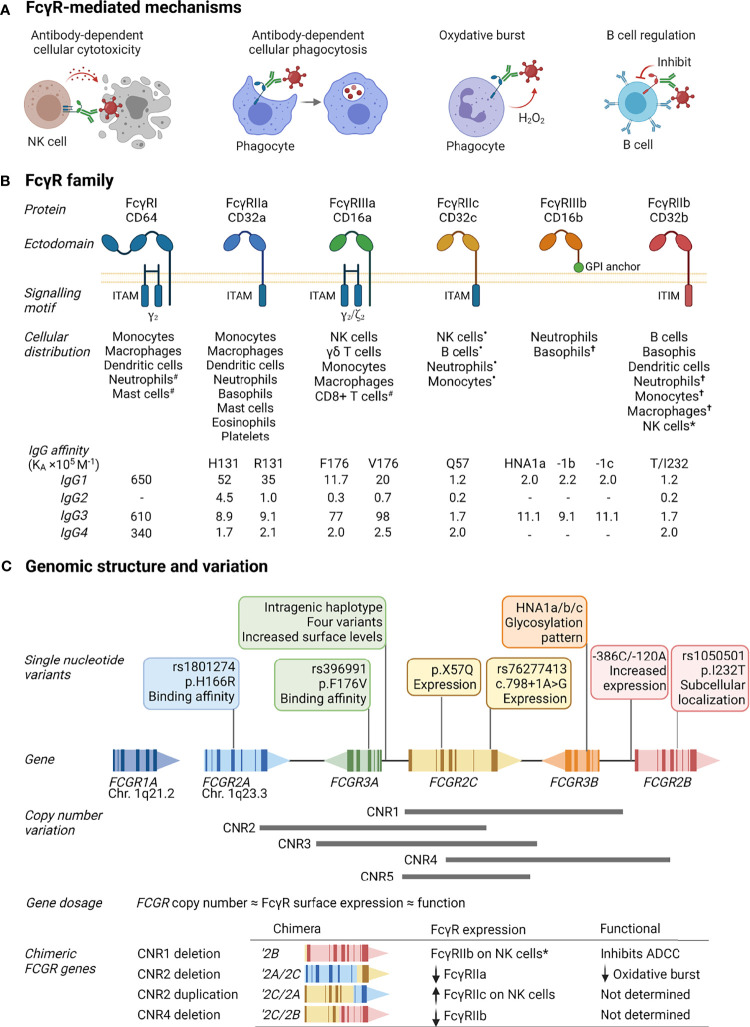
FcγR function, structure and variability. **(A)** FcγRs activate or inhibit immune mechanisms that include killing of infected cells through antibody-dependent cellular cytotoxicity, clearance of immune complexes through phagocytosis, release of reactive oxygen species [superoxide anion (O^-^_2_) and hydrogen peroxide (H_2_O_2_)], and regulation of B cell activation through co-engaging the B cell receptor and inhibitory FcγRIIb by immune complexes. **(B)** FcγRs comprise a family of receptors: FcγRI, FcγRIIa, FcγRIIb, FcγRIIc, FcγRIIIa, FcγRIIIb; also known by their cluster of differentiation (CD) markers CD64, CD32a, CD32b, CD32c, CD16a, and CD16b, respectively. The FcγRs IgG binding chain activates or regulates immune responses depending on its association with or inclusion of an immunoreceptor tyrosine activation motif (ITAM) or inhibitory motif (ITIM). Unique among FcγRs, FcγRIIIb attaches to the cell membrane with a glycosylphosphatidylinositol (GPI) anchor. Each receptor has a specific cell expression profile and affinity for IgG and its subtypes (IgG1-4), shown as affinity constants (K_A_×10^5^ M^-1^); -, no binding. Expression patterns: ^#^inducible expression; ^•^in individuals bearing the *FCGR2C* expression variants (20); **^†^** very low expression or expressed by rare subsets; *expressed in individuals bearing a *FCGR2C-FCGR3B* gene deletion. **(C)** The cluster of *FCGR2A/B/C* and *FCGR3A/B* genes on chromosome 1q23.3 that encode FcγRIIa/b/c and FcγRIIIa/b are polymorphic. Variants include nonsynonymous single nucleotide polymorphisms that alter the receptor’s binding affinity for certain IgG subtypes, determine expression of an otherwise pseudogene, increase surface expression, glycosylation, and subcellular localization. Large segmental duplications and deletions in the *FCGR* cluster further modify FcγR expression levels and create chimeric genes that yield FcγRs with altered cellular distribution and/or function. Created with BioRender.com.

IgG, elicited through active immunization (infection or vaccination) or transferred passively (intravenous infusion or transplacental), modulates an antiviral response through several mechanisms. The antigen binding fragment (Fab) may neutralize virus infection by binding viral surface proteins and preventing attachment to host receptors, while the antibody Fc domain direct immune mechanisms through the engagement of FcγRs. Cross-linking of FcγRs on the cell surface through multivalent interactions, initiates responses that include antibody-dependent cellular cytotoxicity (ADCC), antibody-dependent cellular phagocytosis (ADCP), oxidative burst, release of inflammatory mediators, and regulation of antibody production (**Figure 1A**) (21–24).

FcγRs are a complex family of activating and inhibitory receptors, comprising three classes of molecules and different isoforms: FcγRIa, FcγRIIa/b/c, and FcγRIIIa/b (**Figure 1B**). All FcγRs are glycoproteins belonging to the Ig superfamily and consist of a ligand-binding α-chain with two (FcγRII and FcγRIII) or three (FcγRI) extracellular Ig-like domains, a transmembrane domain, and intracytoplasmic domain. The activating or inhibitory signaling motifs are located either within the α-chain (FcγRII) or associated signaling subunits (FcγRI and FcγRIIIa) (25). Unique to the FcγR family, FcγRIIIb attaches to the cell membrane with a glycosylphosphatidylinositol anchor. Despite lacking intrinsic cytoplasmic signaling domains, FcγRIIIb induces several cell responses (26–28). Each FcγR is expressed on specific cell types, either constitutively or induced, and has particular affinities for IgG and its subtypes (IgG1-4). The genes that encode FcγRs – *FCGR1A*, *FCGR2A/B/C*, and *FCGR3A/B* – are further subject to considerable allelic variation, resulting from segmental genomic duplications/deletions or single nucleotide polymorphisms.

*FCGR2C*, *FCGR3A*, and *FCGR3B* occur at different gene copies due to the gain or loss of defined copy number regions (CNR1-5, **Figure 1C**). The number of *FCGR* genes per diploid genome directly correlate with FcγR surface density and function (29, 30). In addition to this gene dosage effect, duplications/deletions create chimeric *FCGRs* that alter the cellular distribution, expression, and function of FcγRs. A deletion of CNR1, present in 7.4-18.1% of individuals depending on ethnicity, juxtaposes the 5’-regulatory sequences of *FCGR2C* with the coding sequence of *FCGR2B*, creating the chimeric *FCGR2B’* and expression of FcγRIIb on cytotoxic NK cells where it inhibits cell activation and ADCC (31, 32). A CNR2 deletion, present in <1.5% of individuals, leads to an *FCGR2A/2C* chimera that result in reduced FcγRIIa surface levels and oxidative burst response (32, 33). Conversely, a CNR2 duplication, present in 1.6-4.5% of individuals, leads to an *FCGR2C/2A* chimeric gene that increases FcγRIIc expression levels.

Allelic variation for FcγRI is low. In contrast, several single nucleotide variants with a known phenotypic or functional consequence exist for FcγRIIa/b/c and FcγRIIIa/b (34). Distinct amino acid changes in the membrane proximal Ig-like domain of FcγRIIa and FcγRIIIa alter their affinity for IgG subtypes and associated effector functions, including FcγRIIa-p.H166R (alias H131R, rs1801274) and FcγRIIIa-p.F176V (alias F158V, rs396991) (35–38). Conversely, in the transmembrane domain of FcγRIIb, the p.I232T variant (rs1050501) alters its inclusion in lipid rafts and inhibitory signaling (39). In FcγRIIIb, a combination of six amino acid changes determine the human neutrophil antigens (HNA) 1a/b/c – molecules that are antigenically distinct and modulate neutrophil phagocytosis and oxidative burst (40). Unlike other *FCGR*s, *FCGR2C* occurs predominantly as a pseudogene, where a combination of *FCGR2C* minor alleles – p.X57Q (alias X13Q) and c.798+1A>G (rs76277413) – determine its surface expression (20, 41). Other co-inherited single nucleotide variants (haplotypes) within the promotor region of *FCGR2B/C* and spanning *FCGR3A* modulate surface expression levels of FcγRIIb/c and FcγRIIIa, respectively (42–44).

Over the past few years, research identified several new *FCGR* variants of clinical relevance in the context of HIV-1 (described below). Although, linkage disequilibrium (co-occurring variants) in the *FCGR* locus has impeded identification of potential causal variants (19, 45, 46). Studying *FCGR* variants in different population groups in the same and/or different context may help define a role for specific variants, since linkage disequilibrium is inconsistent between geographical populations (19). Of note, describing new *FCGR* variants and assigning them to specific FcγRs warrants caution, since high nucleotide sequence homology between *FCGR*s could lead to inaccurate assignment of variants to specific genes (34); thus, highlighting the need for validated genotyping methods. In general, for the description of new and conventional *FCGR* variants, we encourage the use of a single international genotypic variation nomenclature as described by the Human Genome Variation Society (HGVS) to enable cross-referencing of *FCGR* variants between studies (34, 47). We include here the HGVS name for all variants.

## FcγR Gene Variants and HIV Vaccine Efficacy Trials

**Figure 2:**
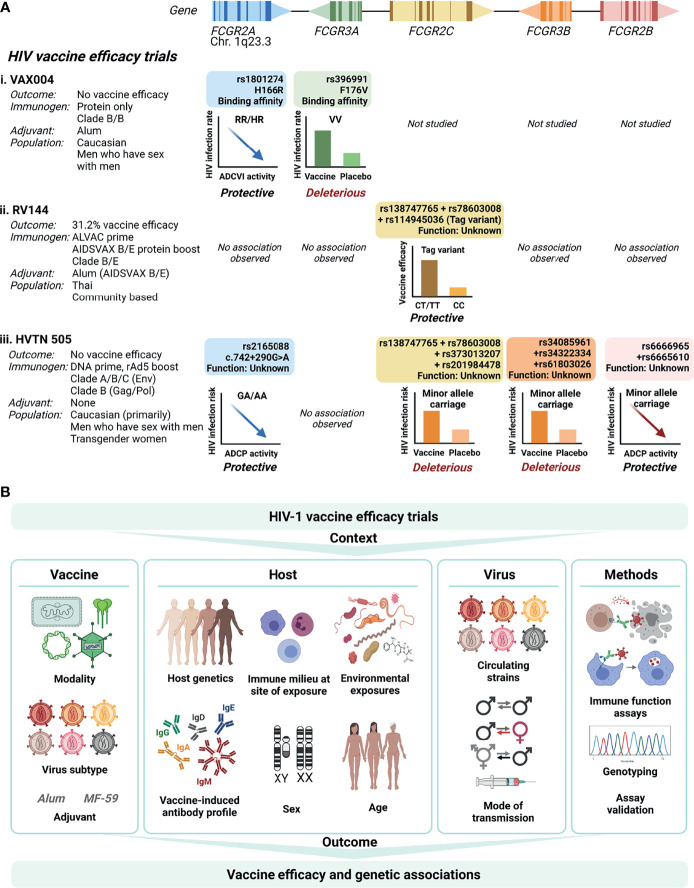
*FCGR* variant associations with HIV-1 vaccine efficacy trial outcomes. **(A)** To date, three HIV-1 vaccine efficacy trials investigated the association between *FCGR* variants and HIV-1 acquisition risk: VAX004, RV144 and HVTN505. The trials differed with regard to vaccine modalities, target HIV-1 subtypes, study populations, mode of HIV-1 transmission, and host ethnicities. In VAX004 and HVTN505 vaccinees bearing minor alleles within *FCGR2A* and *FCGR2B*, enhanced Fc-mediated effector functions [antibody-dependent cellular viral inhibition (ADCVI) and antibody-dependent cellular phagocytosis (ADCP), respectively] associated with reduced risk of HIV-1 acquisition. In VAX004, enhanced HIV-1 acquisition occurred in vaccinees homozygous for the FcγRIIIa-176V allele. Co-inherited intragenic minor alleles in *FCGR2C* enhanced vaccine efficacy in RV144, but increased HIV-1 acquisition risk in HVTN505. Similarly, co-inherited minor alleles in the 5’ untranslated region of *FCGR3B* associated with HIV-1 acquisition risk in HVTN505. **(B)** Defining *FCGR* genetic associations with HIV-1 vaccine efficacy is affected by several factors relating to the vaccine, the host, the virus and methodology used. Created with BioRender.com.

In HIV-1 vaccine efficacy trials, studies have shown clear associations between FcγR-mediated effector functions and risk of HIV-1 acquisition following vaccination (9, 15, 16, 48). To dissect further, three vaccine efficacy studies to date have investigated FcγR variation as a modifier of antibody Fc-mediated effector functions and HIV-1 acquisition risk (**Figure 2A**).

The VAX004 trial evaluated a recombinant envelope protein (AIDSVAX B/B) prime-boost regimen in predominantly Caucasian men who have sex with men (3). Vaccine recipients who remained uninfected had higher antibody-dependent cell-mediated virus inhibition (ADCVI) responses, which encompass ADCC, ADCP and the induction of soluble antiviral factors, than those who became infected (48). The magnitude of ADCVI responses inversely correlated with the HIV-1 acquisition rate, but only in individuals bearing low affinity alleles for FcγRIIa-p.H166R (HR/RR genotypes) and FcγRIIIa-p.F176V (FF genotype) (48) (**Figure 2Ai**). When adjusted for linkage disequilibrium between the two variants, an independent association with FcγRIIa-p.H166R remained. However, the FcγRIIa-p.H166R variant itself did not predict acquisition rate (16). Conversely, in the low risk behavioral group, vaccinees homozygous for the p.176V allele were at greater rate of acquiring HIV-1 compared to those who received the placebo (hazard ratio 4.51), suggesting enhanced infection from the use of AIDSVAX B/B in this genotype group (16).

The RV144 trial, which evaluated a heterologous ALVAC-HIV (vCP1521) canary pox vector prime and AIDSVAX B/E protein boost regimen, demonstrated modest vaccine efficacy (31.2%) in Thai individuals (1). The primary determinants of vaccine efficacy were binding IgG to the variable loops 1 and 2 (V1V2) region of gp120 and binding of plasma IgA to envelope (9). In a secondary analysis, the combination of high levels of ADCC and low plasma anti-HIV-1 envelope IgA antibodies inversely correlated with HIV-1 acquisition risk (9). Variants within FcγRIIIa, the major FcγR involved in NK cell-mediated ADCC, did not associate with HIV-1 acquisition risk (17) (**Figure 2Aii**). Conversely, three single nucleotide variants within *FCGR2C* significantly modified vaccine efficacy that include *FCGR2C* 126C>T (HVGS name: c.134-96C>T, rs114945036), c.353C>T (p. T118I, rs138747765), and c.391+111G>A (rs78603008) (17). All variants were in complete linkage disequilibrium in Thai RV144 trial participants, forming a haplotype. Possession of the haplotype associated with an estimated vaccine efficacy of 91% against CRF01_AE 169K HIV-1 and 64% against any HIV-1 strain, compared to 15% and 11% in the absence of the haplotype, respectively. The functional significance of the variant is unrelated to FcγRIIc surface expression, since only one study participant carried an FcγRIIc-p.57Q allele that predicts expression (17). Alternatively, the haplotype locates within a weak transcriptional enhancer (49). The minor alleles likely abrogate binding of repressor proteins within the regulatory motif and increase mRNA expression. Indeed, in Epstein-Barr virus transformed lymphoblastoid B-cell lines from European Caucasians, the minor allele haplotype associated with increased expression of *FCGR2A* and/or *FCGR2C* exon 7 (50). Other *FCGR2C* variants in complete linkage disequilibrium with the haplotype include c.113-1058T>C (rs2169052/rs115953596) and c.113-684C>T (rs111828362) (49) were not genotyped in RV144 participants and warrant further investigation. Of significance, two components of the haplotype, p.T118I (rs138747765) and c.391+111G>A (rs78603008), are rarely polymorphic in Africans (19), where the RV144 follow-up trial HVTN 702 failed to protect against HIV-1 infection (7).

The HVTN 505 trial that evaluated another heterologous prime-boost regimen – a multigene, multiclade DNA prime and recombinant adenovirus 5 (rAd5) boost – did not show any efficacy in a cohort of predominantly Caucasian men who have sex with men (6). However, ADCP responses and binding of immune complexes to recombinant FcγRIIa-p.166H inversely correlated with HIV-1 acquisition risk (15) (**Figure 2Aiii**). The associations increased for individuals without HIV-1 envelope IgA. Intriguingly, in a phase IIa clinical trial of the same DNA/rAd5 regimen (HVTN 204) (51), a different group did not detect ADCP responses (52). The cause of the distinct observations is unclear; both groups used the same assay albeit a different antibody source (isolated IgG vs. serum) and antigen (vaccine clade-specific gp120 vs. Con S gp140) (52). In the HVTN 505 trial participants, targeted sequencing of regions encoding the extracellular domains of FcγRs identified several variants that associated with HIV-1 acquisition risk or Fc-mediated effector functions. An *FCGR2A* intronic variant modified HIV-1 acquisition risk, *FCGR2A*-intron13-645-G/A (HGVS name: c.742+290G>A, rs2165088) (15). In vaccine recipients bearing the minor allele of c.742+290G>A, the magnitude of ADCP responses and FcγRIIa-p.166H binding to antibody-rgp140 complexes associated with reduced risk of HIV-1 acquisition (15). The functional consequence of *FCGR2A* c.742+290G>A is unknown and it does not appear to be in complete or high linkage disequilibrium with other variants in, or flanking, *FCGR2A.* Inverse correlations between ADCP with HIV-1 acquisition risk similarly occurred for participants bearing minor alleles of two *FCGR2B* variants (synonymous *FCGR2B*-exon5-523-G/A; HGVS name: c.336G>A, rs6665610 and *FCGR2B*-intron14-352-T/G; HGVS name: c.760+26T>G, rs6666965) (18). c.336G>A is in high linkage disequilibrium with seven other *FCGR2B* variants and associated with decreased expression of *FCGR2A* (18).

Furthermore, in HVTN 505 participants, a four-variant *FCGR2C* haplotype and three-variant *FCGR3B* haplotype associated with increased HIV-1 acquisition risk (hazard ratio 9.79 and 2.78, respectively) (18) (**Figure 2Aiii**). The *FCGR2C* haplotype comprise two of the three *FCGR2C* variants identified as protective in the RV144 vaccine trial (p.T118I, rs138747765; and c.391+111G>A, rs78603008). The lack of association with the third *FCGR2C* variant (c.134-96C>T, rs114945036) is likely due to incomplete linkage disequilibrium of the three *FCGR2C* variants in Caucasians (49), the predominant ethnicity of HVTN 505 participants. Additional *FCGR2C* variants were in complete linkage disequilibrium in HVTN 505 participants, *FCGR2C*-intron15-403-C/T (HGVS name: c.760+81C>T, rs373013207] and *FCGR2C*-intron15-433-G/A (HGVS name: c.760+111G>A, rs201984478). The functional consequences of these variants remains to be determined. The haplotype within *FCGR3B* that also associated with increased HIV-1 acquisition comprise three variants in the 5’ untranslated region of *FCGR3B*, 111 to 126 nucleotides upstream of the transcription start site and potentially in the gene promoter region. These include *FCGR3B*-5’utr222-G/A (HGVS name: c.-111G>T; rs34085961), *FCGR3B*-5’utr44-T/A (HGVS name: c.-181T>A, rs34322334), and *FCGR3B*-5’utr99-C/G (HGVS name: c.-126C>G, rs61803026). In individuals with the *FCGR3B* haplotype, vaccination was less likely to induce potentially protective envelope-specific IgG and/or CD8+ T-cell responses than for individuals without the *FCGR3B* haplotype.

## FcγR Variants in Other HIV Infection and Disease Contexts

*Mother-to-child-transmission.* Investigations of *FCGR* variants and mother-to-child-transmission risk are limited to two Kenyan cohorts and one South African cohort (53–55). In a Kenyan cohort of grouped perinatal HIV-1 transmission routes (*in utero*, intrapartum, and breastfeeding), infants with the FcγRIIa-p.166HH genotype were at increased risk of acquiring HIV-1 compared with infants bearing the p.166HR genotype (53). Studies of a Kenyan cohort with a large representation of breastfeeding HIV-1 transmission and our South African cohort with predominantly *in utero* and intrapartum HIV-1 transmission, did not replicate these findings (55, 56). In the latter two cohorts, the maternal FcγRIIIa-F176V variant associated with HIV-1 transmission, although with contrasting findings. In the Kenyan cohort of predominantly breastfeeding women, heterozygous mothers (FV) had an increased risk of transmitting HIV-1 compared to homozygous mothers (combined FF/VV); however, carriage of the 176V allele did not predict HIV-1 transmission (56). If adjustment for multiple comparisons were applied in the study, the association would not have been statistically significant. In contrast, our South African cohort revealed a protective role for the 176V allele in *in utero* transmission, where the association remained significant after adjustment for multiple comparisons (55). A recent study of *FCGR2C* variability in South African children revealed a protective role for a single gene copy of *FCGR2C/3B* per diploid genome (57). In contrast, children bearing the minor allele of the *FCGR2C* variant c.134-96C>T (rs114945036) – identified as protective in Thai RV144 vaccine recipients (17) – were more likely to acquire HIV-1 compared to children homozygous for the c.134-96C allele (57).

*Disease progression.* The FcγRIIa low affinity genotype, p.166RR, predicted a faster CD4 decline compared to p.166RH/HH in the Multicenter AIDS Cohort Study (MACS) of predominantly Caucasian men who have sex with men (58). A similar analysis in Kenyan women – a different host genetic background, sex and route of transmission – showed no effect (59). In addition, the variant did not modify natural control of HIV-1 infection in African Americans (60, 61). Despite convincing evidence for a role for ADCC in natural HIV-1 control [reviewed in (62)], the FcγRIIIa-p.F176V variant does not appear to modify HIV-1 disease course in Caucasians (58) or African Americans (60) (after adjusting for multiple comparisons). Neither FcγRIIa-p.H166R, FcγRIIIa-p.F176V, nor FcγRIIb-p.I232T associated with HIV-1 control in the French multicentric CODEX cohort (63). Of note, the potential for FcγR variants to modify HIV-1 control may only become apparent when considering variability within the ligand, such as IgG γ chain phenotypes (GM allotypes). For example, in individuals bearing the FcγRIIa p.166HH or FcγRIIIa p.176FV/VV genotypes, HIV-1 viraemic control was more likely in the absence of the IgG GM21 allotype (61). Beyond the protein-coding region, a variant located 3.1 kilobases upstream of *FCGR2A*, g.1954 A>G (rs10800309), modified HIV-1 disease progression in a cohort of predominantly Caucasian men and women (63). In particular, homozygosity for g.1954A allele, which associates with increased FcγRIIa surface expression on myeloid cells, predicted natural control of HIV-1 independent of HLA-B57 and HLA-B27 (63). Another non-coding variant, the *FCGR2C* variant c.134-96C>T (rs114945036), predicted HIV-1 disease progression in South Africans (49), the same population where the RV144 follow-on trial, HVTN702, failed to show efficacy (7). However, in the French multicentric CODEX cohort of predominantly Caucasian individuals, the same *FCGR2C* variant did not associate with disease progression (63). It is unclear whether the different outcomes of RV144 and HVTN702 result from diverse population genetics, that include *FCGR2C*, or vaccine-associated factors that include differences in HIV-1 subtype envelopes, mismatched circulating strains, adjuvant or additional booster vaccination. Regardless, the collective findings further emphasize the importance of the *FCGR2C* locus, and additional study in different contexts will help elucidate the underlying protective/deleterious mechanisms.

## Discussion

Many factors affect the host immunological response to immunization and to the pathogen (HIV) encountered. These include i) the route of inoculation and of HIV-1 acquisition, ii) immunogen/virus variability, iii) vaccine regimen (modality, dose, timing, adjuvant), iv) other prior exposures (related or unrelated), comorbidities and pre-existing infections, v) age, vi) sex, vii) geography (population genetics), and viii) genetic variation of the host (**Figure 2B**). The immune milieu present at antigen encounter is affected by all these factors, which collectively define what could be called “an immunological founder effect” – a measure of an individual’s immune capability that dictates the likelihood of producing a protective response to vaccination or infection. As context matters, the antibody Fc-FcγR axis, implicated in protection from acquisition of HIV-1 in vaccine recipients, would be expected to be modulated by these factors.

Investigations of FcγRs and their variants warrant several considerations. i) There are no association studies of *FCGR* copy number variation and HIV vaccine outcome. In RV144, ADCC was a correlate of protection. It is therefore plausible that a CNR1 deletion, which results in the expression of the inhibitory FcγRIIb on NK cells and subsequent inhibition of ADCC, may have an effect on vaccine efficacy. ii) Investigations of single nucleotide variants need to adjust for *FCGR* gene copy number. Certain minor alleles are more prevalent in individuals with more than two gene copies and may confound quantitative trait loci studies of *FCGR* variants (49). iii) Investigations of Fc-mediated effector functions should consider the autologous FcγR variants since they modulate binding of the receptor to antibodies, surface expression levels of the receptor, and/or cell activation/inhibition (64). iv) *FCGR* genes are highly homologous. Assigning single nucleotide variants to specific *FCGR*s requires validated methods. v) Considerable linkage disequilibrium between single nucleotide variants exist across the *FCGR* gene region (19, 45, 46), complicating identification of potential causal variants. vi) Increasing evidence suggest a clinical significance for non-coding *FCGR* variants highlighting potential complex cis- or transgene regulation that warrants characterization and investigation in other contexts. vii) FcγRs often co-occur on the same cell type. Elucidating the role of a single variant requires adjusting for allelic variants in co-expressed FcγRs, since the collective function of all co-expressed FcγRs will determine the effector response. Furthermore, phenotypic and functional analyses of *FCGR* genotype combinations are highly relevant, as demonstrated by an association of the *FCGR2A* rs1801274:rs10800309 diplotype with cell-type specific FcγRII expression (65) and FcγRIIa: FcγRIIIb haplotypes with neutrophil function (66). viii) *FCGR* variation – gene copy number variation, single nucleotide variants, and linkage disequilibrium – differ significantly between population groups and genetic association cannot necessarily be extrapolated between groups. ix) Phenotypic and functional consequences of allelic variants should be studied in the disease context and immune milieu of the condition under study, since disease may alter allelic function (67).

In summary, *FCGR* genetic variants have been associated with protective or deleterious infection and disease outcomes. Much insight can be gained into the potential functional significance of these variants by testing samples from other efficacy trials. For example, HVTN 702, which was non-efficacious in South Africans immunized with subtype C envelope ALVAC-HIV (vCP2438) prime and an MF59-adjuvanted subtype C bivalent envelope protein boost (7). Similarly, individuals passively immunized with broadly neutralizing antibody (VRC01) in the Antibody Mediated Prevention (AMP) trials (68) provide another informative study model. Harnessing host genetic variation between populations, and studying the collective contribution of *FCGR* variants in different infection/disease contexts, will provide much needed insights into what constitutes protective immunity to HIV-1. Importantly, the considerations discussed here extend beyond the context of HIV, bearing relevance to other infections and vaccination strategies that encompass endemic [e.g. malaria (69)], epidemic [e.g. influenza and respiratory syncytial virus (70–72)], pandemic [e.g. severe acute respiratory syndrome coronavirus 2 (SARS-CoV-2) (73)], and emerging/re-emerging infectious diseases [e.g. Ebola (74, 75)].

## Data Availability Statement

The original contributions presented in the study are included in the article/supplementary material. Further inquiries can be directed to the corresponding author.

## Author Contributions

RL and CT conceptualized and wrote the article. Figures were generated by RL. All authors contributed to the article and approved the submitted version.

## Funding

CT receives funding as part of the South African Research Chairs Initiative of the Department of Science and Innovation and National Research Foundation (84177).

## Conflict of Interest

The authors declare that the research was conducted in the absence of any commercial or financial relationships that could be construed as a potential conflict of interest.

## Publisher’s Note

All claims expressed in this article are solely those of the authors and do not necessarily represent those of their affiliated organizations, or those of the publisher, the editors and the reviewers. Any product that may be evaluated in this article, or claim that may be made by its manufacturer, is not guaranteed or endorsed by the publisher.
